# Dielectrophoresis for Bioparticle Manipulation

**DOI:** 10.3390/ijms151018281

**Published:** 2014-10-10

**Authors:** Cheng Qian, Haibo Huang, Liguo Chen, Xiangpeng Li, Zunbiao Ge, Tao Chen, Zhan Yang, Lining Sun

**Affiliations:** Robotics and Microsystems Center, College of Mechanical and Electrical Engineering & Collaborative Innovation Center of Suzhou Nano Science and Technology, Soochow University, Suzhou 215000, China; E-Mails: ffsyccc@163.com (C.Q.); chenliguo@suda.edu.cn (L.C.); licool@mail.ustc.edu.cn (X.L.); gezunbiao@hotmail.com (Z.G.); cht22@sina.com (T.C.); yangzhan@suda.edu.cn (Z.Y.); lnsun@hit.edu.cn (L.S.)

**Keywords:** dielectrophoresis, lab-on-a-chip, bioparticle, trapping, detection, focusing, pairing, separation

## Abstract

As an ideal method to manipulate biological particles, the dielectrophoresis (DEP) technique has been widely used in clinical diagnosis, disease treatment, drug development, immunoassays, cell sorting, *etc.* This review summarizes the research in the field of bioparticle manipulation based on DEP techniques. Firstly, the basic principle of DEP and its classical theories are introduced in brief; Secondly, a detailed introduction on the DEP technique used for bioparticle manipulation is presented, in which the applications are classified into five fields: capturing bioparticles to specific regions, focusing bioparticles in the sample, characterizing biomolecular interaction and detecting microorganism, pairing cells for electrofusion and separating different kinds of bioparticles; Thirdly, the effect of DEP on bioparticle viability is analyzed; Finally, the DEP techniques are summarized and future trends in bioparticle manipulation are suggested.

## 1. Introduction

In recent decades, the optical [1,2], microfluidic [3], mechanical [4], magnetic [5] and electrical fields have been widely applied in controlling micro-/nano-particles, such as trapping, focusing, characterizing, paring and separating particles. The precise control of particles, especially cells, viruses, proteins, DNA molecules and other biological particles, has widespread application prospects in biomedical [6], bioscience [7] and other fields [8].

The different particle control methods are compared in Table 1. As shown in the table, electrical fields are very suitable for bioparticle manipulation with the advantages of strong controllability, easy operation, high efficiency and slight damage to targets. Two of the most important electrokinetic phenomena are electrophoresis [9,10] (EP) and dielectrophoresis (DEP). DEP is an ideal particle control method for electrically neutral particles. When a dielectric particle is suspended in the electric field, it will be polarized into dipoles, and Coulomb interaction will exist between the dipoles and the electric field. If the electric field is non-uniform, a net force will act on the particle to drive the particle to move toward/against the direction of the electric field maxima [11]. Reuss F firstly discovered the phenomenon of DEP in the study of clay particles. After that, Pohl conducted a series of research studies on the phenomenon of DEP [12,13,14], and he named the phenomenon “dielectrophoresis”. With the development of precision machining technology [15], DEP technology has gained rapid development since 1990. In recent years, with the development of Micro-Electro-Mechanical System (MEMS) technology, micro-/nano-scale microelectrodes can be more easily integrated into the chip, which makes the DEP technique able to be combined with other functions of lab-on-a-chip. DEP has advantages, such as high control efficiency, easy operation and equipment miniaturization. Hence, it has been widely used in biomedical, bioscience and other fields [16,17].

**Table 1:** The comparison of different particle control methods.

Methods	Controllability	Operation	Efficiency	Cost	Damage
optical	strong	hard	low	high	slight
microfluidic	weak	easy	high	low	little
mechanical	strong	hard	low	low	large
magnetic	strong	hard	low	low	slight
electrical fields	strong	easy	high	low	slight

## 2. Theory and Model

Dielectrophoresis can be divided into conventional dielectrophoresis (cDEP), traveling-wave dielectrophoresis (twDEP) and electrorotation (ROT).

### 2.1. Calculation Model

#### 2.1.1. Conventional Dielectrophoresis

In 1978, Pohl firstly established the approximate model of conventional dielectrophoresis on the basis of the classical Maxwell electromagnetic field theory:

(1) $$F_{\text{DEP}}=2\text{π}r^{3}{\text{ε}}_{\text{m}}\text{Re}\left[f_{\text{CM}}\right]\nabla E^{2}$$

where *r* is the radius of a spherical particle, ${\text{ε}}_{\text{m}}$ is the absolute permittivity of the media, $E^{2}$ is the rms value of the electric field, $\text{Re}\left[f_{\text{CM}}\right]$ is the real part of the Clausius–Mossotti (CM) factor, which refers to positive and negative dielectrophoresis (pDEP and nDEP), and $f_{\text{CM}}$ is given by:

(2) $$f_{\text{CM}}=\frac{{\text{ε}}_{\text{p}}^{\text{*}}-{\text{ε}}_{\text{m}}^{\text{*}}}{{\text{ε}}_{\text{p}}^{\text{*}}+2{\text{ε}}_{\text{m}}^{\text{*}}}$$

(3) $$\text{Re}\left[f_{\text{CM}}\right]=\frac{\left({\text{σ}}_{\text{p}}-{\text{σ}}_{\text{m}}\right)\left({\text{σ}}_{\text{p}}+2{\text{σ}}_{\text{m}}\right)+{\text{ω}}^{2}\left({\text{ε}}_{\text{p}}-{\text{ε}}_{\text{m}}\right)\left({\text{ε}}_{\text{p}}+2{\text{ε}}_{\text{m}}\right)}{{\left({\text{σ}}_{\text{p}}+2{\text{σ}}_{\text{m}}\right)}^{2}+{\text{ω}}^{2}{\left({\text{ε}}_{\text{p}}+2{\text{ε}}_{\text{m}}\right)}^{2}}$$

where ${\text{ε}}_{\text{p}}$ is the absolute permittivity of a particle, ${\text{σ}}_{\text{m}}$ and ${\text{σ}}_{\text{p}}$ are the conductivity of the medium and particle. ${\text{ε}}_{\text{m}}^{\text{*}}$ and ${\text{ε}}_{\text{p}}^{\text{*}}$ are the complex permittivities of the medium and particle, which are related to the conductivity σ and the angular frequency ω of the electric field:

(4) $${\text{ε}}^{\text{*}}=\text{ε}-i\frac{\text{σ}}{\text{ω}}$$

#### 2.1.2. Electrorotation

If a particle is suspended in an electric field that rotates at an angular velocity equal to the angular frequency ω, it will experience a rotational torque. In 1988, Arnold established the model of electrorotation based on the model of conventional dielectrophoresis [18]:

(5) $$T_{\text{ROT}}=-4\text{π}r^{3}{\text{ε}}_{\text{m}}\text{Im}\left[f_{\text{CM}}\right]E^{2}$$

where $T_{\text{ROT}}$ is the time average torque, and $\text{Im}\left[f_{\text{CM}}\right]$ is the imaginary part of Clausius–Mossotti factor:

(6) $$\text{Im}\left[f_{\text{CM}}\right]=\frac{-3\text{ω}\left({\text{ε}}_{\text{m}}{\text{σ}}_{\text{p}}-{\text{ε}}_{\text{p}}{\text{σ}}_{\text{m}}\right)}{{\text{ω}}^{2}{\left({\text{ε}}_{\text{p}}+{\text{ε}}_{\text{m}}\right)}^{2}+{\left({\text{σ}}_{\text{p}}+2{\text{σ}}_{\text{m}}\right)}^{2}}$$

#### 2.1.3. Traveling-Wave Dielectrophoresis

A traveling-wave electric field will be produced if the phase-shifted voltages are applied to the parallel electrodes. The particle subjected to the traveling-wave field will move along or against the direction of filed travel. In 1992, Huang established the model of traveling wave dielectrophoresis [19]:

(7) $$F_{\text{TWD}}=\frac{-4{\text{π}}^{2}r^{3}{\text{ε}}_{\text{m}}\text{Im}\left[f_{\text{CM}}\right]E^{2}}{\text{λ}}$$

where λ is the wavelength of the electric field. It’s equal to the distance between electrodes of the same phase.

#### 2.1.4. Unified Model

In the traveling wave electric field, particles experience both DEP force and the twDEP force [20]. It is very important to have a generalized theory. By summing up the former dielectrophoresis mode, Wang established the unified model of conventional dielectrophoresis and traveling wave dielectrophoresis [21]:

(8) $$F=2\text{π}r^{3}{\text{ε}}_{\text{m}}\left\{\text{Re}\left[f_{\text{CM}}\right]\nabla E^{2}+\text{Im}\left[f_{\text{CM}}\right]\left(E_{\text{x}}^{2}\nabla {\text{φ}}_{\text{x}}+E_{\text{y}}^{2}\nabla {\text{φ}}_{\text{y}}+E_{\text{z}}^{2}\nabla {\text{φ}}_{\text{z}}\right)\right\}$$

where ${\text{φ}}_{\text{i}}$ is the phase component in Cartesian coordinate.

### 2.2. Particle Model

**Figure 1:**
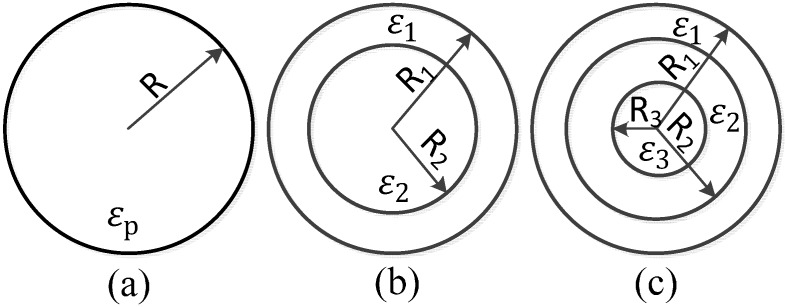
Spherical model. (**a**) Single-shell; (**b**) dual-shell; (**c**) multi-shell.

All of the theories above are based on a single-shell spherical model, as shown in Figure 1a. However, most of the bioparticles are complex, heterogeneous structures with a cell membrane, cytoplasm and nucleus. Each part has different electrical properties. In this situation, the single-shell spherical model does not work for it, and the multi-shell spherical model should be used to account for these differences [22], as shown in Figure 1b,c. After a series of calculations, the multi-shell model can be equivalent to a single-shell model [23].

As shown in Figure 1b, the radius of the outermost layer is $R_{1},$ the radius of the inner layer is $R_{2},$ and the permittivity of each layer corresponding to $R_{1},R_{2}$ is $\varepsilon_{1},\varepsilon_{2}$ (when the alternating electric field is applied, the complex permittivity must be used.). The equivalent permittivity is given by:

(9) $${\text{ε}}_{\text{p}}=\;{\text{ε}}_{1}\frac{{\left(\frac{R_{1}}{R_{2}}\right)}^{2}+\;2\left(\frac{{\text{ε}}_{2}-{\text{ε}}_{1}}{{\text{ε}}_{2}+2{\text{ε}}_{1}}\right)}{{\left(\frac{R_{1}}{R_{2}}\right)}^{2}-\left(\frac{{\text{ε}}_{2}-{\text{ε}}_{1}}{{\text{ε}}_{2}+2{\text{ε}}_{1}}\right)}$$

From inside out, the multi-shell model in Figure 1c can be equivalent to a single-shell model through the two-step calculation of Equation (9).

The spherical model is very simple and common, but most of the bioparticles are not spherical, they are closer to ellipsoid. The calculation method of DEP force on the ellipsoid model is very complicated, which has been introduced in detail by Yang *et al.* [24].

## 3. Technology and Application

In recent years, DEP has made great developments in bioscience. The basic bioparticle capture is extensively used in biology and biomedical applications such as cell injection [25], cell transfer [26,27], *in vitro* fertilization [28], dielectric characterization [29,30], *etc.* The efficiency and accuracy of the biosensor are improved after being integrated with the DEP chip [31,32], since DEP attracts bioparticles to the detection area or even enhances the contact between bioparticles and immobilized antibodies [33]. DEP can also be used for sample preparation [34], molecular enrichment [35], and biological assays [36,37,38]. Compared with the conventional sample preparation, DEP simplifies the enrichment steps and improves the efficiency of sample preparation. For cell fusion, the DEP technique can be used to improve the pairing precision of cells, which has made great contributions to crossbreeding [39], cancer immunotherapy [40], biomedical research [41,42] and other fields. Besides, the DEP separation technique has been used widely in the purification of vaccine and protein [43,44], cancer diagnosis [45] and cell sorting [46,47], which lays a solid foundation for the diagnosis and treatment of disease [48]. From basic cell capture to clinical diagnosis of disease, from cell collection to sample preparation, from cell separation to the purification of vaccine and protein, DEP has shown a great potential for development in bioscience [49,50].

### 3.1. Trapping

The trapping of bioparticles has important applications in terms of cell injection, cell transfer, *in vitro* fertilization, cell interaction, stem cell research and immunoassays.

#### 3.1.1. Microelectrode

The DEP force acting on the particle mainly depends on its size and the gradient of the square of the magnitude of the electric field. Therefore, for a bioparticle with a small size, the DEP force is very weak, which makes trapping of the particle difficult. One method to increase the DEP force acting on the bioparticle is to improve the gradient of the square of the magnitude of the electric field by enhancing the strength of the electric field. With the development of MEMS technology, the scale of microelectrodes integrated into the chip is getting smaller and smaller. As a result, the electric field strength provided by low voltage is so strong that the range of the controllable particle size is expanding gradually. For example, Hölzel R *et al.* trapped single protein molecules of *R*-phycoerythrin with an electrode gap of 500 nm [51], and Kumemura M *et al.* successfully trapped DNA molecules with a microelectrode [52].

#### 3.1.2. Insulator-Based Dielectrophoresis

In addition to reducing the electrode gap, insulating microstructures can enhance the strength of the electric field as well, which is known as insulator-based dielectrophoresis (iDEP). As compared to traditional electrode-based dielectrophoresis, in an iDEP device, external electrodes are employed to generate a uniform electric field, and insulating microstructures are embedded in the microchannel to squeeze the electric field. Thereby, a high electric field gradient with a local maximum is created. It has the advantages that the structure is mechanically robust and chemically inert, and a very high electric field may be applied without gas evolution, due to electrolysis at metal DEP electrodes. Besides, electrode-based dielectrophoresis devices use small amplitude AC signals, while high amplitude DC voltages are usual directly applied to iDEP devices, which can be used to pump the sample solution via electrokinetic flow. Chou CF *et al.* used blocks to squeeze electric field iDEP to trap and concentrate single and double-stranded DNA [53]. An open-top microchannel was adapted in his work to make the design easier and faster for sample cleaning. Besides, the Joule heating effect was suppressed, because the heat could be dissipated more quickly. Lapizco-Encinas *et al.* used insulating posts to create field nonuniformities in the channel [54]. By increasing the conductivity and lowering the pH of the medium, he successfully trapped BSA protein with nDEP at lower electric field strengths. Furthermore, the negative effect of the electric field on protein was reduced, since the protein was trapped by nDEP. This demonstrated the great potential of iDEP as a technique for bioparticle manipulation. Moreover, other forms of electrodes [55,56] were used to create field nonuniformities in the DC-iDEP devices as well.

There are a few works in the literature in which AC signals are applied in iDEP devices. Jen CP *et al.* designed a chip for cell trapping with open-top microstructures [57]. The open-top microstructures have great visibility for observation during the experiment, and they are suitable for trapping biological samples that can undergo further treatment easily. Moreover, the open-top microstructures are beneficial to dissipate the Joule heat quickly. In a later study, he found that live HeLa cells exhibited nDEP at low frequencies of 100 Hz to 1 kHz and exhibited pDEP at high frequencies of 1 MHz, but dead HeLa cells exhibited pDEP at all the frequencies applied. Therefore, Jen CP successfully achieved selective trapping of dead HeLa cells from live cells at a frequency of 1 kHz [58].

#### 3.1.3. Adjustable Trapping Position

**Figure 2:**
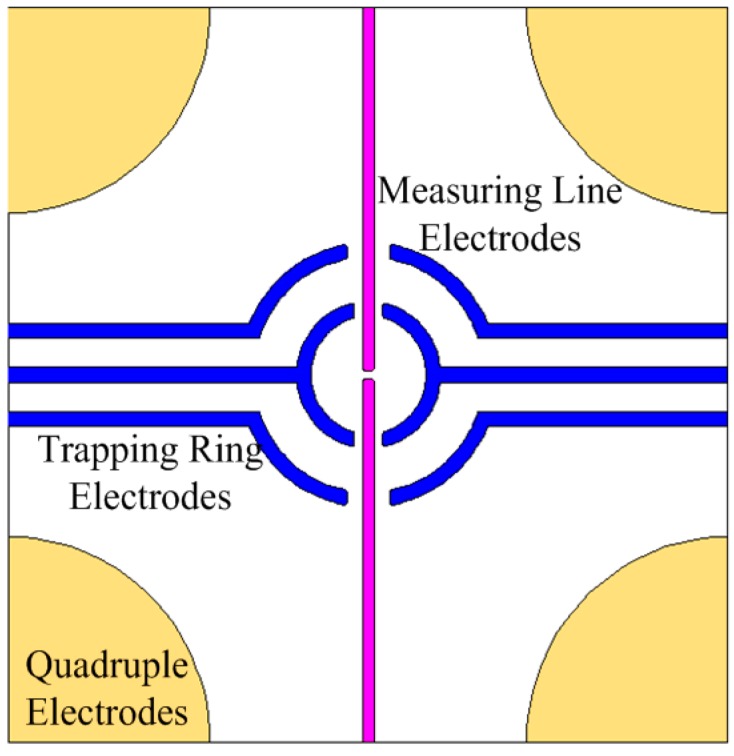
Schematic diagram of the adjustable trapping position chip [61].

This plays a vital role in biological measurement and medical research to analyze single cells, bacteria and viruses. Particles should be trapped in the detection region before analysis, while the position of the particle has a big impact on the accuracy of the measurement results. pDEP, iDEP and other trapping methods have been used to trap single biological particle, but the trapped position generated from the same structure is fixed [59,60]. Wang CC *et al.* developed a single-cell trapping chip, and its trapping position is adjustable [61]. In the chip, a plane structure is designed to generate an adjustable trapping position by utilizing the voltage phase-controlled method and nDEP effect. As shown in Figure 2, the red lines are measuring electrodes that influence the position of the captured cell, and the blue lines are symmetrical arc geometry trapping electrodes that influence the strength of the nDEP force for capturing cell in the capture region. There are three modes to manipulate cells: trapping mode, releasing mode and adjustable trapping mode. The trapping mode can generate a fixed trap position to capture the particle; the releasing mode is able to move the cell away from the trapped position; and the adjustable trapping mode is able to generate an unfixed trap position, so that the position of the trapped cell is adjustable. In this chip, the trapped vertical position can be adjusted in a range from 0 to 26.4 μm, and the capture height can be adjusted in a range from 0 to 45 μm, which significantly improves the accuracy of the measurement results.

#### 3.1.4. Dielectrophoresis Tweezers

**Figure 3:**
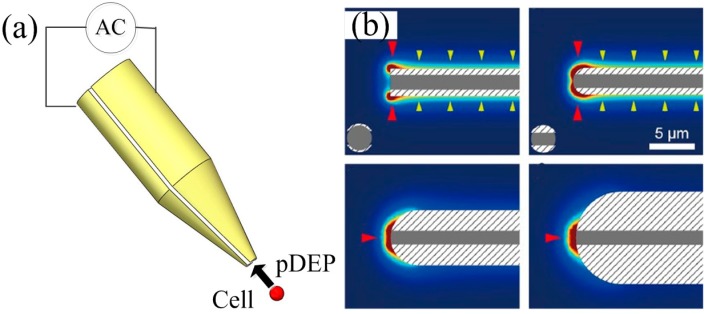
DEP tweezers. (**a**) Schematic diagram of DEP tweezers designed by Hunt TP; (**b**) the electric field strength simulation [63].

For the fixed electrode DEP chip, it’s difficult to adjust the manipulation position. Although Wang CC *et al.* have developed an adjustable trapping position chip, the adjustment range is still quite small. DEP tweezers can position a single cell in three dimensions, or transfer a single cell to any designated areas. As shown in Figure 3a, Hunt TP *et al.* developed the DEP tweezers using pDEP force to hold single cell at the end of a micromanipulator [62]. The tweezers are made by a glass rod and a pair of thin gold-film electrodes. When the voltage is applied on the electrodes, a strong non-uniform electric field will form at the needle tip. If a certain cell is close to the needle tip, it will be attracted to the field maximum at the tip of the electrodes. Hunt TP trapped a yeast cell for many hours using DEP tweezers, and the yeast cell bred daughter cells in 2 h and became a cell mass with many cells within 6 h. This demonstrated that the strong electric field at the tip of the tweezers did not harm the yeast cells.

In later research, Kodama T *et al.* found that the cylindrical-tip tweezers were not suitable for single cell manipulation [63]. He simulated the squared electric field strength of the round-tipped tweezers compared with the cylindrical-tip shape by using three dimensional models. As shown in Figure 3b, for the cylindrical-tip tweezers, there are two strong electric field areas at the outer edge of the tweezers, which is not suitable for single-cell manipulation. A round-tip with a radius of 3 mm is the most suitable for single-cell manipulation within the simulated geometries, because the electric field focuses on the center of the tweezers’ tip. Kodama T separated fluorescently labeled cells and non-labeled cells successfully using round-tipped tweezers. This demonstrated that the round-tip DEP tweezers were able to selectively manipulate target cells one by one.

### 3.2. Detection

With the continuous development of economic globalization and food culture diversity, food hygiene and safety have strongly gotten people’s attention. Bacterial content in food and drink is an important index in evaluating food safety. Impedance measurement technology is used to detect the bacterial content, but the traditional impedance measurement technique is time-consuming and has low sensitivity in the case of a lower sample concentration, because it takes a long time to trap enough targets to detection an area to obtain a significant electrical signal. One of the effective strategies to enhance sensitivity and to reduce the testing time is to integrate a DEP chip into the impedance measurement system [64]. Dielectrophoresis impedance measurement (DEPIM) has advantages, such as high efficiency, high sensitive and low costs [65,66].

**Figure 4:**
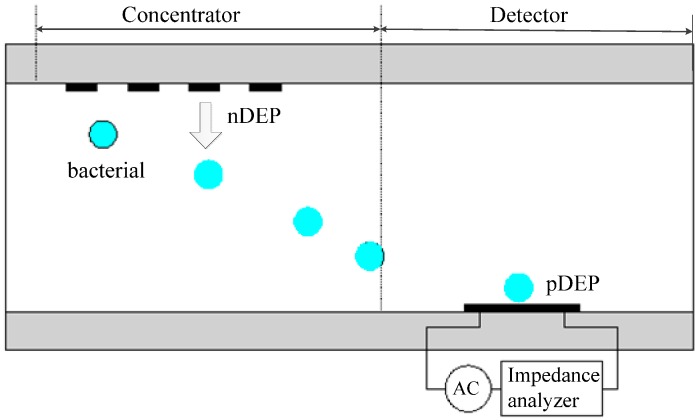
Schematic diagram of the bacterial detection chip based on nDEP and pDEP [69].

Yang L *et al.* proposed a biochip for foodborne bacteria detection [67]. It is proven in his experiments that the biochip integrated with the DEP technique has higher detection efficiency. Menachery A *et al.* developed a biochip for bioparticle detection [68]. The biochip used twDEP force to drive bioparticles to the detection area for detecting. Hamada R *et al.* designed a bacteria detection chip utilizing both pDEP and nDEP to enhance the trapping efficiency [69]. As shown in Figure 4, there are two working areas on this chip: the concentration area and the detection area. When the bacteria pass through the concentration area, they will move towards the bottom of the channel under the nDEP force. After entering the detection area, the bacteria are captured at the detector electrode by pDEP force. Hamada R found that nDEP concentrator can further improve the DEP trapping efficiency compared with the detector only using pDEP.

DEPIM can be used in immunoassay as well. The antibodies are immobilized onto the electrode surface to serve as the ligand for the target bioparticles. After antigen-antibody binding, the impedance between electrodes changes due to the immune response, which is measured by the impedance sensor. Then, the relevant information of the bioparticles is acquired by analyzing the impedance variation. DEP technology can dramatically enhance the antibody capture efficiency because DEP attracts bioparticles to the chip surface and enhances the contact between bioparticles and immobilized antibodies [70].

It is very important to quantify protein–protein interactions, protein–glycan interactions, protein–molecule interactions and other biomolecular interactions. Bashir *et al.* used the DEP technique to characterize biomolecular interaction [71]. In the microfluidic device, functionalized beads are initially bound on the functionalized surface. They are pulled away from the surface by DEP force. As a result, the interactions between molecules and beads can be quantitatively examined by analyzing the unbinding voltages. This study provides a simple and robust manner to characterize biomolecular interactions, and it can be used to measure the mechanical properties of bioparticles as well. In a later study, Bashir *et al.* improved the device towards a wide range of loading rates [72]. Forces across the range of force loading rates (10^−4^ to 10^4^ pN/s) were tested in their experiments, and the results proved that the device could be used for precise and simultaneous examination of various molecular interactions.

### 3.3. Focusing

Apart from trapping, DEP force can also be applied to focusing particles. DEP focusing technology has shown great value in sample preparation, molecular enrichment and biological laboratories [73,74].

**Figure 5:**
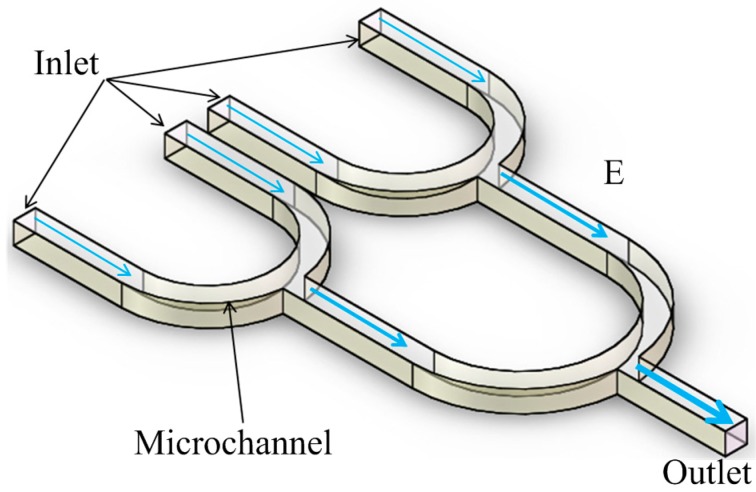
An insulating tree structure for electric field focusing [76].

As shown in Figure 5, Chen DF *et al.* developed a microfluidic device for rapid and efficient enrichment of particles with direct current DEP [75,76]. Just like iDEP, in this concentrator, external electrodes are employed to generate a uniform electric field in the microchannel, and an insulating tree structure is used for multilevel electric field focusing. The electric field gradually becomes strong from the left side (inlet) to the right side (outlet). Under the experimental conditions, both the electrokinetic effect and dielectrophoresis simultaneously take place within the concentrator at different regions. The electric field is relatively lower on the left side and particles move to the right side mainly under the electrokinetic effect, whereas the electric field is strong on the right side, such that particles are trapped by DEP forces and focused in the right side. Because the insulating tree structure uses multilevel electric field focusing, it only requires a low voltage for particle enrichment, and it has the advantage of high enrichment efficiency.

With the advantages of being mechanically robust, chemically inert and have a low cost, the iDEP technique has been widely used for particle enrichment as well [77]. Usually, DC voltages are applied to iDEP devices [78]. However, the DC-iDEP technique has the drawback that it requires high voltages, which causes a serious Joule heating effect and affects the viability of bioparticles [79]. By both experiments and simulations, Zhu J *et al.* demonstrated that using a DC-biased AC electric field can significantly improve particle focusing performance as compared to a pure DC electric field [80]. Lewpiriyawong N [81] tested the trapping voltage for concentrating 15-μm particles under a pure DC voltage of 3400 V and a DC-offset AC voltage of 30 + 635 sin(2π(5 kHz) *t*) V. Experimental results showed that the total DC-offset AC electric field strength required to concentrate 15-μm particles was significantly reduced to 85.9%, as compared to using a pure DC electric field. Besides, Joule heating effect was significantly suppressed under the DC-offset AC electric field. Therefore, the DC-offset AC electric field is more suitable for bioparticle enrichment.

### 3.4. Pairing

Cell fusion is the core technology of cell engineering. It has been widely applied in crossbreeding [39], cancer immunotherapy [40], biomedical research [41,42] and other fields. The most important step in cell fusion is accurate cell pairing, and the DEP technique has been used to improve the pairing precision of cells. Compared with biological, chemical and other fusion methods, electrofusion has the advantages of being label-free, easy operation and low toxicity [82,83].

**Figure 6:**
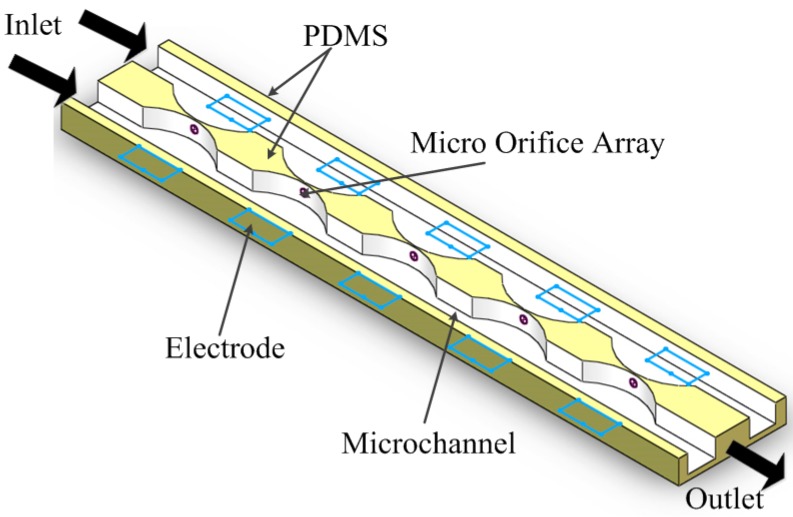
Schematic diagram of electrofusion chip designed by Gel.

However, since traditional electrofusion is achieved by electrode-based dielectrophoresis directly [84], the heterogeneous cell pairing efficiency is low due to the random cell contact, and it is difficult to avoid multi-cell fusion. With the development of microfabrication techniques, insulating microstructures are integrated into chips to improve heterogeneous cell pairing efficiency. In the electrofusion chip, insulating microstructures are not only used as the partition structures, but are also used to trap and pair cells based on iDEP. Gel M *et al.* designed a micro-orifice-based fusion chip [85], as shown in Figure 6. The channel is divided into two parts by the wall, on which orifices are aligned in a linear manner along the channel. Two different kinds of cells enter into the microchannel from the opposite sides, and they are attracted to the micro-orifices by pDEP force; therefore, different cells can make contact with each other. However, the throughput of this chip is not very high, because the orifices can only be aligned one-dimensionally along the channel.

In a later study, Kimura Y *et al.* improved the chip by using a sheet on which orifices are arranged in a flat manner [86]. There are 6000 orifices on the sheet compared to only 80 when aligned in a linear manner; hence, the throughput can be improved greatly. However, since a high electric field gradient only exists near the micro-orifices, cells in the lower channel can’t be lifted to the micro-orifices. Hence, the device needs to be flipped over while keeping the DEP voltage on to keep cells in the upper channel trapped. After that, cells in the upper chamber now move toward the micro-orifices under gravity and will be trapped by pDEP force. Therefore, the two kinds of cells can make contact with each other.

Şen M *et al.* fabricated a device for single-cell pairing based on a combination of nDEP and pDEP [87]. By applying a voltage to the left interdigitated array electrodes, one kind of cell is trapped on the left side of the microwell under the pDEP force, while the other one is trapped on the right side of the microwell in the same way. Subsequently, they are pushed together by using the nDEP to achieve cell pairing.

The heterologous cell pairing based on the partition structure is very efficient. However, the cell sizes are limited by the diameter of the micro-orifices; in addition, the fusion cells will be subject to extrusion stresses when passing through the micro-orifices, which may affect the cell viability.

### 3.5. Separation

Particle separation has a wide range of applications in bioscience. The DEP technique has been used for bioparticle separation, and it has aroused wide concern among people. The DEP separation technique offers the possibility for the diagnosis of disease [88,89].

#### 3.5.1. Time-Based Separation

The DEP particle separation can be divided into two categories: time-based separation and space-based separation. In time-based separation, one type of particle is trapped by the pDEP force, while the other one is suspended in the solution under nDEP force; and the one that is suspended can be separated first. The early studies of DEP traps have given insight into the method of time-based particle separation. For example, Voldman J *et al.* tested how well DEP traps could hold beads against fluid flows [90]. Li H *et al.* built a finite element model to study the holding forces for both pDEP and nDEP traps [91]. Furthermore, he measured the voltages necessary to capture different particles against fluid flows, which has made a fundamental contribution to developing particle separation devices.

Morgan H *et al.* proposed a polynomial electrode to separate tobacco mosaic virus (TMV) and herpes simplex virus (HSV) [92]. In the device, HSV is repelled to the center of the electrode array by nDEP force, while TMV is trapped at the electrode edges under pDEP force, resulting in the separation of two types of particle. Gascoyne P *et al.* achieved isolating malaria-infected cells from blood with spiral electrodes [93]. Four phase AC signals are applied on the spiral electrodes. Normal erythrocytes in the device are trapped at electrode edges by pDEP force, while parasitized cells are levitated under nDEP force and simultaneously carried towards the center of the spiral electrodes under twDEP force. Therefore, normal erythrocytes and parasitized cells are separated. Li H *et al.* used interdigitated electrodes to separate live and dead *Listeria* cells [94]. In his separation experiments, most live cells were trapped at the edges of electrodes, while the dead cells were repelled to the top centers of electrodes, and the separation efficiency was as high as about 90%. Arnold WM *et al.* achieved live and dead yeast cell separation with the “headlands and bays” electrodes [95]. The live yeast cells are attracted to the regions of the maximum field, while the dead ones are repelled to the regions of the minimum field, resulting in the separation of live and dead yeast cells.

Choi W *et al.* proposed a castellated electrode for bioparticle separation. The gradient of the square of the magnitude of the electric field reaches a maximum in the vicinity of the electrodes, and particles experiencing pDEP force will be attached to the electrodes. With this chip, Choi W achieved healthy and non-healthy oocyte separation, which would be helpful for *in vitro* fertilization [96]. Yu L *et al.* developed a DEP field-flow separation chip with a 3D electrode structure. He simulated the flow field in the microchannel defined by electrodes with the semicircular, triangular and rectangular geometries. He found that the semicircular-shaped electrode could generate a specific fluid velocity gradient, which is suitable for particle separation [97]. In the chip, the cells experiencing pDEP force are attached to the convex areas of the electrode, and others are repelled to the concave areas of the electrode under nDEP force. According to the simulation of the flow field in the microchannel, there are fluidic dead zones in the concave areas, while the fluid velocity reaches its maximum in the center of convex areas. The cells trapped by pDEP are swept out of the microchannel first by hydrodynamic force. After the electric field is removed, the other cells will be swept out. As we can see, time-based DEP separation is mainly aimed at the particles that have different real parts of the Clausius–Mossotti factor.

**Figure 7:**
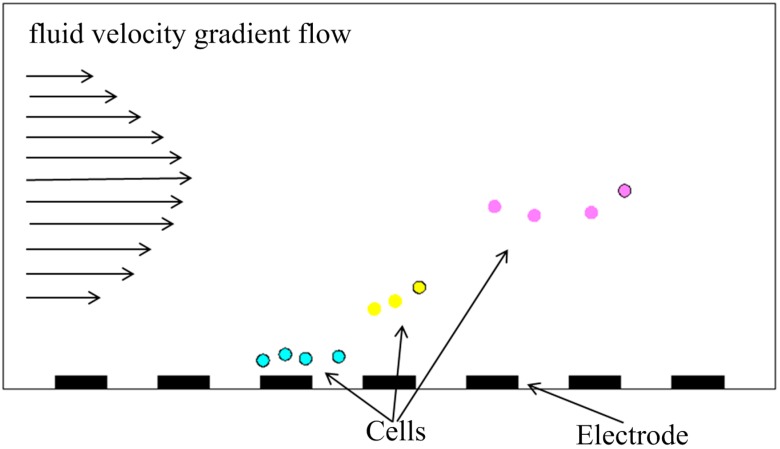
Schematic diagram of DEP field-flow fractionation.

Dielectrophoresis field-flow fractionation (DEP-FFF) is another kind of time-based separation method, and the advantage of this method is that more than two kinds of particles can be separated in this way [98]. As shown in Figure 7, three different kinds of particles are suspended in solution under the combination of nDEP force and gravity, and they are suspended at different heights because of the difference between particle dielectric properties. There is a fluid velocity gradient flow in the channel, so that particles of different heights are carried at different velocities and can be separated successfully. Gascoyne P *et al.* successfully isolated MDA-435, MDA-468, and MDA-231 cells from blood samples containing up to 28 × 10^6^ PBMN cells by DEP-FFF [99]. Vykoukal J *et al.* isolated putative stem cells from adipose tissue using DEP-FFF with success. The work of both Gascoyne P and Vykoukal J *et al.* demonstrated a potential clinical application for DEP-FFF [35].

#### 3.5.2. Space-Based Separation

**Figure 8:**
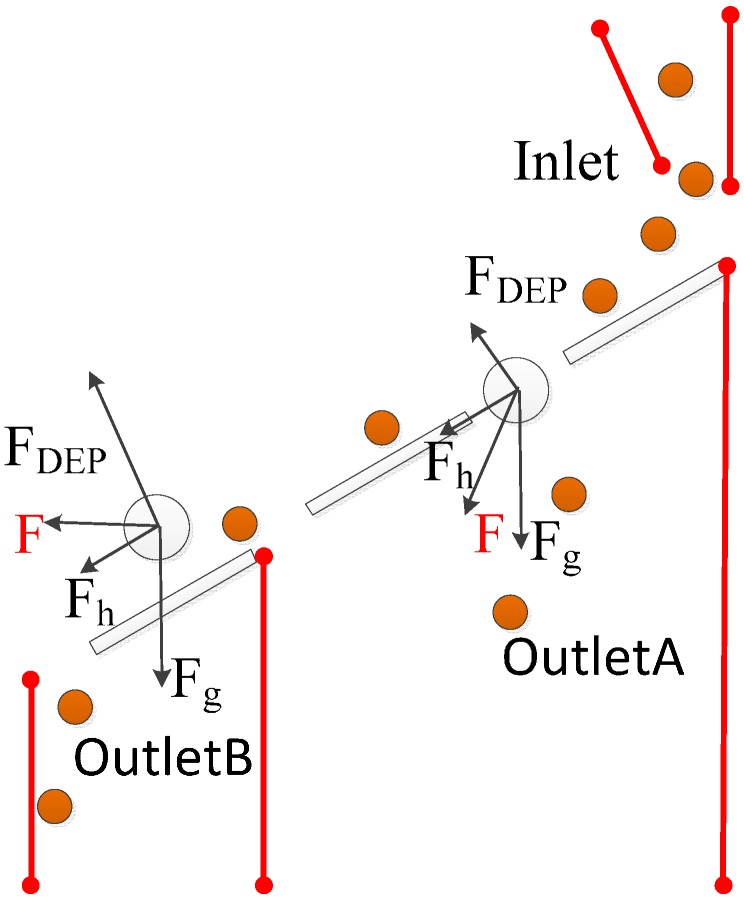
The simulation analyses of forces on particles.

In space-based separation, particles are suspended in different positions under the combination of nDEP force, gravity or other forces. Therefore, different kinds of particles can be separated from different outlets at the same time. This means that space-based separation takes less time to carry all of the particles out of the channel than time-based separation. In addition, the negative effects of the electric field on bioparticle viability are reduced in space-based separation, since particles are away from the electrodes. Therefore, it’s a very efficient way to achieve bioparticle separation. In the separation chip designed by Piacentini N, hydrodynamic force combined with nDEP force is used to separate particles with respect to their size difference [100]. Platelets and blood cells enter into the microchannel from the left inlet. The bigger red blood cells and white ones are pushed to the right side of the channel under nDEP force and collected at the right outlet, while smaller platelets at the left, because nDEP force is not strong enough to push the platelets to the right side when the particles are flowing through the channel. It’s a very efficient separation of platelets from other blood cells. Lee J *et al.* developed a high throughput cell sorting chip based on gravitational, hydrodynamic and nDEP forces [101]. The chip doesn’t need an external pump, because the flow is driven by gravity. After the sample is focused in the focusing channel, it enters into the separation channel. As shown in Figure 8, the direction of nDEP force is perpendicular to the electrode plane and pointing upward, while the composite force of gravity and hydrodynamic force is pointing downwards at an angle. If the nDEP force isn’t strong enough, the composite force of gravitational, hydrodynamic and nDEP force is pointing downwards. As a result, particles will be settled down. On the other hand, if the nDEP force is strong enough, the composite force is pointing upwards. As a result, particles will be settled down after they pass through the electrode plane. Therefore, different kinds of particles can be separated by properly adjusting the voltage.

**Figure 9:**
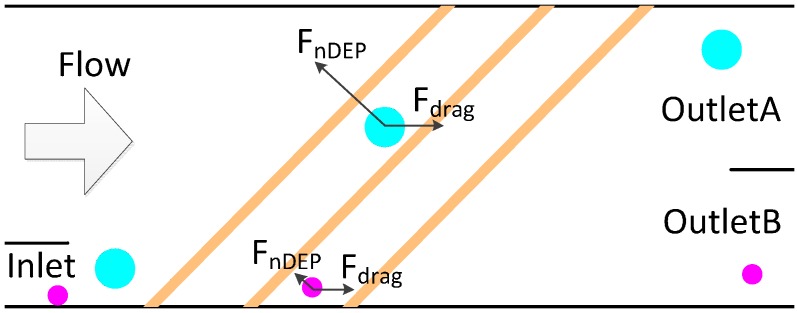
Schematic diagram of lateral separation.

**Figure 10:**
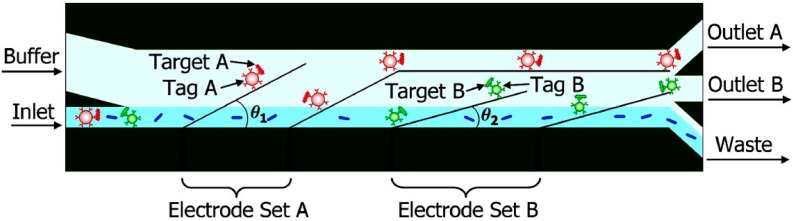
Schematic diagram of the lateral DEP microseparator [104].

If the planar interdigitated electrode array is placed at an angle to the direction of the flow, as shown in Figure 9, particles will have a lateral displacement under nDEP force when passing over the electrode array. The lateral nDEP force can be used for particle separation as well [102,103]. It’s a specific type of space-based separation. The schematic of the multi-target bacterial cell sorting chip designed by Kim U is shown in Figure 10. Two sets of electrode arrays are placed at different angles to the direction of flow. Cells are subjected to a hydrodynamic force and a lateral nDEP force in the horizontal plane when they pass though the electrode array. If the nDEP force exceeds the hydrodynamic force in the direction perpendicular to the electrode array, then the cells will be pushed to the left side of the channel [104]. As shown in Figure 10, target and nontarget cells enter into the microchannel from the inlet. While passing through the electrode Set A, Target A experiencing the bigger nDEP force, is repelled to the left side of the channel and collected at the Outlet A, whereas Target B and nontarget cells experiencing the smaller nDEP force don’t have a lateral displacement. The electrode Set B is placed at a smaller angle than Set A; therefore, the component of nDEP force perpendicular to the direction of flow become larger than before. Target B can be pushed away from the right side and is collected at the Outlet B, whereas the rest of the nontarget cells are collected in the waste. Han S *et al.* developed a biochip to measure the size distribution of blood cells based on a lateral DEP separator [105]. In the separator, there is only one set of electrode arrays, and the flow rate is very low. Therefore, blood cells in different size ranges will have different lateral displacements under nDEP force when passing over the electrode array, and will finally be collected at different locations.

The effect of electric field on cell viability was measured in Kim’s experiments, and the data shows that the viability of cells was not affected by the electric field. One of the reasons is that cells are pushed away from regions of high electric field under nDEP force. Hence, this is a good way to separate bioparticles.

#### 3.5.3. Insulator-Based Separation

With the advantages of being mechanically robust, chemically inert and have low costs, the iDEP technique has been used for particle separation as well. In 2004, Lapizco-Encinas *et al.* demonstrated the concentration and separation of live bacteria in water [106]. An array of insulating posts was microfabricated into the device to produce field nonuniformities in the channel. The bacterial species exhibited different DEP mobilities in the microchannel. The *Escherichia coli* were trapped at the weakest applied electric field, while the *Bacillus* species was trapped at different characteristic threshold fields. As a result, different species of bacteria were selectively trapped and separated. In the same device, Lapizco-Encinas successfully separated live and dead *E. coli* by applying stepped DC voltages [107]. In 2009, Lapizco-Encinas *et al.* demonstrated the concentration and separation of microorganisms, *E. coli* and *Saccharomyces cerevisiae* cells, and they were manipulated by employing nDEP in the microchannel. *E. coli* cells were trapped at a higher electric field (400 V/cm), while *S. cerevisiae* cells were trapped at a lower electric field (200 V/cm). Hence, *E. coli* and *S. cerevisiae* cells were separated selectively.

Jen CP *et al.* arranged the insulating posts array like a series of quadrupoles, and he realized live and dead HeLa cells separation with a combination of hydrodynamic flow and the DEP response of cells [108]. In the device, dead mammalian cells were trapped in high electric field regions, where the flow velocity was lower. However, live cells were repelled to the low electric field regions under the nDEP force, and the flow velocity in those regions was higher. Therefore, live and dead HeLa cells are separated.

Kang Y *et al.* demonstrated the continuous separation of microparticles by size with DC iDEP [109]. The nonuniform electric field is generated by an insulating block fabricated inside the channel, and the electric field strength is strongest at the corners of the block. Particles deviate from the streamline under nDEP force when flowing through the corners of the block, and the degree of deviation depends on the particle size. As a result, the particles of different sizes can be continuously separated. In a later study, Kang Y *et al.* studied the separation of white blood cells and breast cancer cell groups [110]. They found that cancer cells die a short time after they are exposed to a very high electric field. To solve the problem, they modified the design and created a triangular block to reduce the negative effects.

**Figure 11:**
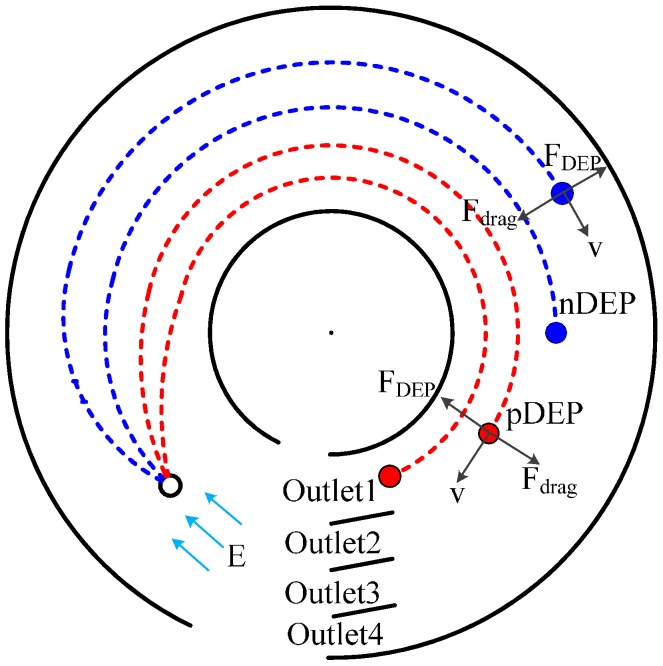
Schematic diagram of iDEP separation in a circular microchannel [111].

Zhang L *et al.* designed the circular channel for continuous particle separation based on iDEP [111], as shown in Figure 11. The nonuniform electric field is generated by applying voltage over the channel. Particles with different dielectric properties enter into the channel from the left side. They move to different locations across the channel under different DEP force. In the meantime, they move towards the outlet along the channel under electro-osmotic flow. In the device, the direction of movement of the particle (moving towards the inner or outer circle) depends on the CM factor. Hence, particles with different dielectric properties will be collected at different outlets.

Viefhues M *et al.* [112,113] embedded the S-shaped ridge in the microchannel to squeeze the electric field. Due to the disadvantages of the pure DC voltage such as a serious Joule heating effect, AC and DC voltages were used to generate electrokinetic transport and dielectrophoresis effects. The particles in the channel far away from the constriction are mainly driven by the electroosmotic fluid flow, while DEP is negligible, because the electric field is almost uniform. When the particles approach the constriction, the nDEP force gets strong. Just like lateral separation, if the nDEP effects are stronger than the linear electrokinetic effects in the direction perpendicular to the constriction, particles will be pushed to the other side of microchannel, otherwise they will pass through the constriction directly.

**Figure 12:**
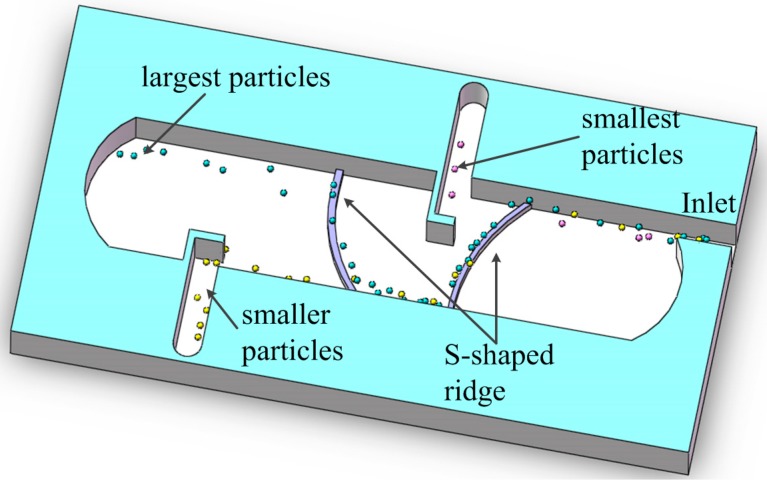
Schematic diagram of the Insulator-based DEP chip for multiple-separation [114].

As shown in Figure 12, there are two constrictions in the microchannel. The smallest particles go across the first constriction directly, and the other two kinds of particles are repelled to the left side. By adjusting the ratio between the linear electrokinetic effects controlled by the DC electric field and the nDEP effects governed by the AC electric field, the smaller particles can go across the second constriction directly, while the largest particles are repelled to the right side. Therefore, three kinds of particles are separated due to their size difference. Viefhues M *et al.* successfully separated three DNA species using this structure [114].

#### 3.5.4. Separation Based on Traveling-Wave Dielectrophoresis

**Figure 13:**
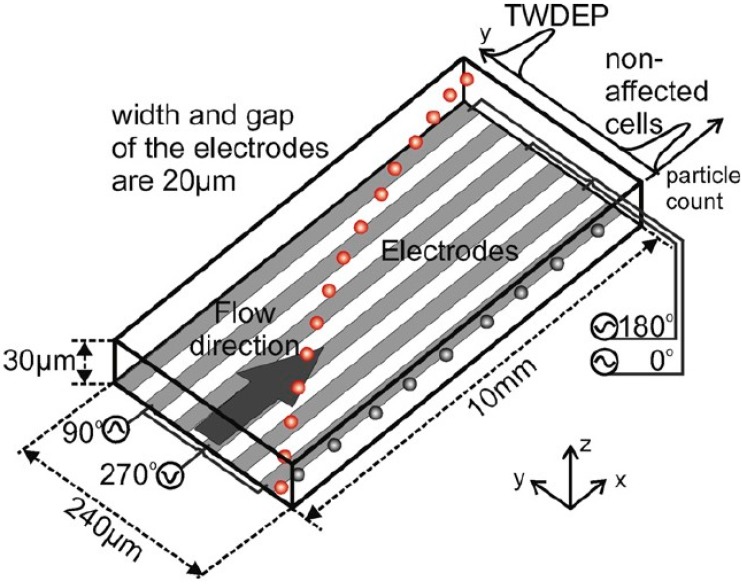
A microchip for biological cells separation base on twDEP [116].

The twDEP can be generated by applying phase-shifted voltages on the parallel electrodes. The particle experiencing twDEP force moves along or against the direction of travel of the field depending on the imaginary part of the Clausius–Mossotti factor [115]. Hence, different particles can be separated due to the difference of the imaginary part of Clausius–Mossotti factor. Driesche S developed a continuous cell separation device based on twDEP, as shown in Figure 13. Phase-shifted voltages of 90° are applied on the parallel electrodes. Jurkat cells $\left(\text{Im}\left(f_{CM}\right)=0.52\right)$ and *Lactobacillus casei* bacteria $\left(\text{Im}\left(f_{CM}\right)=0.01\right)$ are injected at the right side of the channel. Jurkat cells are pushed towards the left side of the channel under twDEP force, whereas bacteria stay on the right side of the channel, because of the weak twDEP force that they suffer. In the meantime, both of the cells and bacteria are pushed towards the outlet by pressure-driven flow. In the end, Jurkat cells are collected on the left side and *L. casei* bacteria are collected at the right side [116].

The high electric field may have a negative effect on cell viability. nDEP force can push particles away from the high electric field. However, for a planar electrode, the farther it is from the electrode, the smaller the twDEP force and the lower the separation efficiency it will have. Cheng IF used the a 3D electrodes to solve this problem [117]. The electrode arrays are arranged on the top and bottom sides of the channel. Particles are repelled to the center of the electrodes by nDEP force; therefore, the twDEP force will not be too weak.

Choi E *et al.* developed a chip for high-throughput particle separation. Along the microelectrode track, the electrode spacing increases [118]. The mixed particles suspend in the solution move towards the direction of travel of the field. With the increase of the electrode spacing, the nDEP force and twDEP force decrease, and the particles will fall into specific regions, depending on their physical parameters. Therefore, particle separation is achieved.

**Figure 14:**
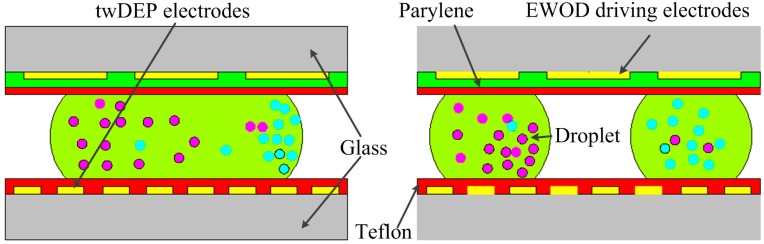
In-droplet particle separation procedures by twDEP and EWOD [119].

twDEP combined with electrowetting-on-dielectric (EWOD) technology has been used for particle separation [119,120]. As shown in Figure 14, first, the particles in the droplet are separated under twDEP force. Then the droplet is split into two daughter droplets under EWOD. Finally, two types of particles are separated into two respective daughter droplets. Furthermore, this technology can be used for particle enrichment. If a mother droplet has only one type of particle, the mother droplet is split into two daughter droplets after the particles are transported to the droplet edge. As a result, particle concentration in the daughter droplets increases. The advantage of this technology is that the chip doesn’t need an external pump and complicated microchannel networks.

#### 3.5.5. Separation Based on Dual Frequency Dielectrophoresis

**Figure 15:**
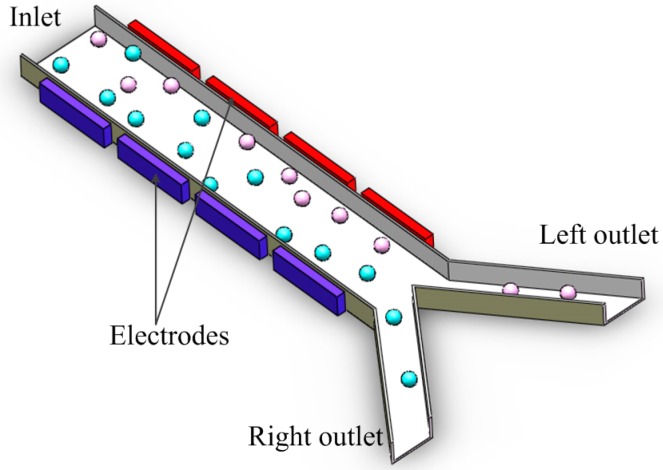
Schematic diagram of the microchip for particle separation based on dual frequency dielectrophoresis.

A schematic of particle separation based on dual frequency dielectrophoresis is shown in Figure 15. The interdigitated electrodes are embedded on both sides of the channel. With different frequencies and magnitudes of signals applied to opposing sets of the electrodes, different kinds of particles can reach balance at different positions in the channel, and they are carried to the outlet by the hydrodynamic force. The separation effect of this chip was tested by Wang L. In the experiments, the frequencies and voltages applied to the left electrodes are 8 V, 10 MHz, and to the right are 10 V, 50 kHz. Both HEK293 cells and polystyrene beads experience nDEP force [121]. Due to the difference in dielectric properties, for the beads, the nDEP force is stronger in the right side than in the left side, whereas, for the cells, nDEP force is stronger in the left side. As a result, the beads are pushed to the left side and the cells are pushed to the right side, so that they can be collected at different outlets.

#### 3.5.6. Isodielectric Separation

The uniqueness of isodielectric separation is that the particles are separated in an electrical conductivity gradient solution [122]. The particles are pushed towards the direction of decreasing medium conductivity under DEP force. When the particles move to the position where the medium conductivity is the same as the particles’, $\text{Re}\left(f_{CM}\right)=0,$ the particles will no longer be subject to DEP force, and they are carried to the outlet by hydrodynamic force. As a result, the particles with different conductivity are separated at different locations. This is an effective way for multi-particle separation.

## 4. The Effect of Dielectrophoresis on Bioparticle Viability

Although DEP technology has been widely used in bioparticle manipulation, it still has some influence on bioparticle viability [123]; if used improperly, it may cause bioparticle death [124]. The effect of DEP on bioparticle viability mainly depends on the electric field strength and the Joule heating effect.

Under pDEP force, bioparticles will move towards the direction of the high electric field. However, the high electric field may cause bioparticle death. Kang Y *et al.* found in the cell separation experiments that live cancer cells die short time after they are exposed to a very high electric field [110]. Nevertheless, Hunt TP trapped yeast cells for many hours under pDEP force, and the yeast cell was still able to grow and divide [62]. The major difference between Kang and Hunt’s experiments is that Kang Y used DC voltage in the experiments, whereas Hunt TP used AC voltage in the experiments.

Hakoda M *et al.* systematically studied the effect of electric field on cell activity [125]. In his experiments, the device was immersed in an ice water tank to control the temperature at 278 K, which could prevent the deactivation of cells due to temperature changes. The effect of the electric field on cell viability was tested in his first set of experiments. After 10 min, the cell concentration did not change under the condition of no electric field. In the DC electric field (10 kV/m), the cell concentration decreased slightly, and the nonviable cells increased. In the AC electric field (1 kHz, 21 kVrms/m), the cell concentration decreased sharply, and the nonviable cells increased. Furthermore, the influence of frequency on the viability of cells was tested, and the result showed that at 1 kHz, the viability decreased dramatically by increasing the electric field strength. However, the viability decreased slightly by increasing the electric field strength at 300 and 1000 kHz, and it was higher than in the DC electric field (10 kV/m). As we can see in the experiments above, high-frequency AC field is more suitable for cell manipulation than a DC electric field or a low-frequency AC field. In addition, the growth activity of cells exposed to the electric field was tested as well. The cells in the first set of experiments were cultivated for 24 and 48 h, and the results were surprising. When the cells exposed to the DC electric field (10 kV/m) were cultivated for 48 h, the nonviable cells increased, and the viable cells did not. On the contrary, in the AC electric field (1 kHz, 21 kVrms/m), the viable cells increased sharply. It could be found that the adverse effect on the cells exposed to the AC electric field by the cultivation is almost lost, whereas the DC electric field does harm to the cell viability. The findings add to the evidence that the AC electric field is a more secure method for cell manipulation than the DC electric field.

Apart from using nDEP force to keep bioparticles away from electrodes, Bashir R *et al.* developed a cell manipulation chip [126], in which a thick glass coverslip was placed directly on the electrodes. As a result, bioparticles won’t make contact with electrodes directly. In the experiment, Bashir R manipulated HeLa cells with pDEP, and the viability of the HeLa cells after DEP manipulation was investigated. The results showed that the cells were still alive and proliferating on the glass coverslip. In addition, it’s worth mentioning that the DEP electrodes can be reused again and again since the electrodes does not make contact with the sample directly.

The exposure time in the electric field is one of the factors that affects cell activity, especially in a high strength electric field. In Donato S’s experiment, cells were trapped under the pDEP for a period of time. The result indicates that the cell metabolism is affected by the electric field after 10 min of exposure, while the cells no longer produce GFP after 15 min of exposure, and most of the cells died [127]. Therefore, for bioparticles, the exposure time in the electric field must be controlled.

The Joule heating effect is another unfavorable factor influencing cell activity. The Joule heating effect can raise the local temperature of the buffer, which may change the structure and properties of the cell, and even cause cell death. Hence, it has great significance for bioparticle manipulation to suppress the Joule heating effect.

High voltage will generate a large power density in the fluid surrounding the electrodes, which could give rise to a large temperature increase in the medium. With the development of MEMS technology, the scale of microelectrodes integrated into the chip is getting smaller and smaller, and as a result, a much smaller applied voltage can be used to manipulate the particles. Since the voltage is reduced, the Joule heating effect is suppressed. In addition, the DC voltage will produce a large amount of Joule heating, while the combination of AC and DC voltages can suppress the Joule heating effect significantly [81]. Besides, the conductivity of the buffer is one of the factors affecting the Joule heating effect. Jaeger MS *et al.* studied the electric field-induced warming in a DEP chip [128]. In the experiments, they found that temperature variation is almost a linear interrelation with the electric conductivity of the solution, and the Joule heating effect can be ignored in the low conductivity solution. However, for bioparticle manipulation, usually a high conductivity buffer must be used. Hence, within the allowable range for bioparticle manipulation, it’s helpful to suppress the joule heating effect by reducing the buffer conductivity.

For a planar electrode, the DEP force decrease sharply with the increased distance from the electrode. Therefore, it’s difficult to manipulate the particles far away from the electrode. One method to solve this problem is to increase the applied voltage, but this will raise the temperature of the buffer due to the Joule heating effect. Hence, it is not conducive for cell manipulation. Another method is to use a 3D electrode, which can suppress the DEP force decay [129]. Moreover, Tay EH found in his research that the Joule heating effect in a chip with a 3D electrode is 8–10 times lower than in the classical planar structure. Therefore, at 3D electrode is more suitable for bioparticle manipulation [130].

## 5. Concluding Remarks

Following the discovery of the DEP, various classical DEP theories were established to explain the phenomenon of DEP. With the rapid development of computer processing power, researchers can develop DEP chips more quickly and easily by numerical simulation.

Over the past decade, DEP technology has been developing rapidly. Two opposite approaches are studied to make DEP more suitable for bioparticle manipulation. One is to reduce the distance between electrodes to be able to reduce the applied voltage. Thanks to the advanced micro-processing technology, micro/nano-scale microelectrodes can be more easily integrated into the chips. As a result, the particle size that can be manipulated by DEP is getting smaller and smaller. In addition, since the electrode gap is decreased, the voltage applied to the electrodes is reduced, and then the Joule heating effect is suppressed. Hence, the bioparticle viability improves. Moreover, a smaller voltage is also conducive to the safety of staff. However, the drawback is gas evolution due to the electrolysis of the metal electrode. The other is to place the electrodes completely outside the region of interest and to generate a nonuniform electric field by an insulating microstructure. The iDEP technique overcomes the disadvantage of a metal electrode, which involves the phenomenon of gas evolution due to electrolysis. However, the applied voltages are very high, which causes a serious Joule heating effect and affects the viability of bioparticles. It’s a challenge to develop a DEP chip with a small applied voltage and good biocompatibility in the future.

The trapping of a bioparticle has important applications in terms of cell injection, cell transfer, *in vitro* fertilization, cell interaction, stem cell research and immunoassays. A DEP chip based on an electrode array or an electric cage can achieve multi-particle capture, but the trapped position generated from the same structure is fixed. Although DEP tweezers can position a cell in three dimensions, and transfer the cell to any targeted areas, it can only operate on a single cell at a time. Hence, the efficiency is relatively low. A more efficient DEP chip that has the function of multi-particle capture and an adjustable trapping position should be developed in the future.

The DEP technique has been widely used for bioparticle characterization and detection, such as bacteria detection, immunoassay, gene monitoring, protein expression, protein purification, real-time PCR, cancer biomarker discovery and biomolecular interaction analysis. Integrating a DEP chip in the impedance measurement system can reduce the testing time. In immunoassay, DEP can dramatically enhance the antibody capture efficiency. Besides, the DEP technique provides a simple and robust manner to characterize biomolecular interactions. In the future, more applications of bioassays based on DEP will be discovered.

DEP focusing technology has shown great value in sample preparation, molecular enrichment, and in biological laboratory. iDEP is used for rapid concentration of particles, as its advantages. Usually, DC voltages are directly applied to iDEP devices through external electrodes. However, high DC voltages are required in iDEP devices, which lead to a serious Joule heating effect, and they affect bioparticle viability. The DC-offset AC electric field is used to solve this problem, because the total DC-offset AC electric field strength required to concentrate the same particles is significantly reduced compared to using the pure DC electric field. Besides, the Joule heating effect is significantly suppressed under the DC-offset AC electric field. Chips for bioparticle concentration should be further improved toward higher efficiency, lower energy consumption, and less effect on the bioparticle.

Cell electrofusion has been widely applied in crossbreeding, cancer immunotherapy, biomedical research and other fields. Usually, traditional electrofusion is achieved by electrode-based dielectrophoresis directly. The heterogeneous cell pairing efficiency is low due to the random cell contact, and it is difficult to avoid multi-cell fusion. With the development of microfabrication techniques, microstructures are integrated into the chips to improve heterogeneous cell pairing efficiency. However, cell viability may be affected by the extrusion stresses from the micro-orifices. Hence, the electrofusion chip with the characteristics of high heterogeneous cell pairing efficiency and little effect on the bioparticle needs to be further studied.

DEP particle separation technology has shown a wide range of research value in cell sorting, disease diagnosis and treatment of cancer. There are many kinds of particle separation methods. Time-based DEP separation is mainly aimed at particles that have different real parts of the Clausius–Mossotti factor. In time-based separation, because the two kinds of particles experience different kinds of DEP forces, one type of particle is trapped, while the other one is suspended in the solution, and then the one suspended in the solution can be separated first. In space-based separation, different kinds of particles are suspended in different positions under the combination of nDEP force, gravity or other forces. Therefore, different kinds of particles can be isolated from different outlets. Lateral DEP separation is mainly aimed at particles that have similar dielectric properties but different sizes. The particles with different imaginary parts of the Clausius–Mossotti factor can be separated by twDEP. In addition, some novel methods, such as dual frequency dielectrophoresis and isodielectric separation, have been used for bioparticle separation as well.

More importantly, the DEP technology should be used carefully for bioparticle manipulation, because the electric field and the Joule heating effect have a negative effect on bioparticle viability. Compared to the DC electric field, the high-frequency AC electric field helps to reduce the damage to bioparticles. The bioparticle survival rate in the high electric field is very low, so pDEP is only suitable to manipulate cells for a short period of time. Miniaturized electrodes and low buffer conductivity help to reduce the Joule heating. Compared to a planar electrode, a 3D electrode can reduce the Joule heating to a large extent, which is more suitable for bioparticle manipulation. However, the machining process of a 3D electrode is relatively complex and costly. Therefore, a DEP chip that is suitable for bioparticle manipulation needs to be further studied.

Along with unremitting scientific progress, dielectrophoresis technology will continue to play a more important role in life sciences.

## References

[1] Shao B., Zlatanovic S., Ozkan M., Birkbeck A.L., Esener S.C. (2006). Manipulation of microspheres and biological cells with multiple agile vcsel traps. Sens. Actuators B.

[2] Huang S.-B., Wu M.-H., Lin Y.-H., Hsieh C.-H., Yang C.-L., Lin H.-C., Tseng C.-P., Lee G.-B. (2013). High-purity and label-free isolation of circulating tumor cells (CTCS) in a microfluidic platform by using optically-induced-dielectrophoretic (ODEP) force. Lab. Chip.

[3] Tanyeri M., Schroeder C.M. (2013). Manipulation and confinement of single particles using fluid flow. Nano Lett..

[4] Lilliehorn T., Simu U., Nilsson M., Almqvist M., Stepinski T., Laurell T., Nilsson J., Johansson S. (2005). Trapping of microparticles in the near field of an ultrasonic transducer. Ultrasonics.

[5] Shah J., Wilkins E. (2003). Electrochemical biosensors for detection of biological warfare agents. Electroanalysis.

[6] Hsiung L.-C., Chiang C.-L., Wang C.-H., Huang Y.-H., Kuo C.-T., Cheng J.-Y., Lin C.-H., Wu V., Chou H.-Y., Jong D.-S. (2011). Dielectrophoresis-based cellular microarray chip for anticancer drug screening in perfusion microenvironments. Lab. Chip.

[7] Laux E.M., Kaletta U.C., Bier F.F., Wenger C., Hölzel R. (2014). Functionality of dielectrophoretically immobilized enzyme molecules. Electrophoresis.

[8] Ivanoff C.S., Hottel T.L., Garcia-Godoy F. (2012). Dielectrophoresis: A model to transport drugs directly into teeth. Electrophoresis.

[9] Ermolina I., Morgan H. (2005). The electrokinetic properties of latex particles: Comparison of electrophoresis and dielectrophoresis. J. Colloid Interface Sci..

[10] Zhao S., Wang J., Ye F., Liu Y.-M. (2008). Determination of uric acid in human urine and serum by capillary electrophoresis with chemiluminescence detection. Anal. Biochem..

[11] Jones T.B., Jones T.B. (2005). Electromechanics of Particles.

[12] Pohl H.A., Crane J.S. (1971). Dielectrophoresis of cells. Biophys. J..

[13] Pohl H.A., Crane J.S. (1972). Dielectrophoretic force. J. Theor. Biol..

[14] Pohl H.A., Pohl H. (1978). Dielectrophoresis: The Behavior of Neutral Matter in Nonuniform Electric Fields.

[15] Verpoorte E., de Rooij N.F. (2003). Microfluidics meets MEMS. Proc. IEEE.

[16] Sebastian A., Buckle A.M., Markx G.H. (2007). Tissue engineering with electric fields: Immobilization of mammalian cells in multilayer aggregates using dielectrophoresis. Biotechnol. Bioeng..

[17] Braff W.A., Willner D., Hugenholtz P., Rabaey K., Buie C.R. (2013). Dielectrophoresis-based discrimination of bacteria at the strain level based on their surface properties. PLoS One.

[18] Arnold W., Zimmermann U. (1988). Electro-rotation: Development of a technique for dielectric measurements on individual cells and particles. J. Electrost..

[19] Huang Y., Wang X.-B., Tame J., Pethig R. (1993). Electrokinetic behaviour of colloidal particles in travelling electric fields: Studies using yeast cells. J. Phys. D.

[20] Cen E.G., Dalton C., Li Y., Adamia S., Pilarski L.M., Kaler K.V. (2004). A combined dielectrophoresis, traveling wave dielectrophoresis and electrorotation microchip for the manipulation and characterization of human malignant cells. J. Microbiol. Methods.

[21] Wang X.-B., Huang Y., Becker F., Gascoyne P. (1994). A unified theory of dielectrophoresis and travelling wave dielectrophoresis. J. Phys. D.

[22] Irimajiri A., Hanai T., Inouye A. (1979). A dielectric theory of “multi-stratified shell” model with its application to a lymphoma cell. J. Theor. Biol..

[23] Huang Y., Holzel R., Pethig R., Wang X.-B. (1992). Differences in the AC electrodynamics of viable and non-viable yeast cells determined through combined dielectrophoresis and electrorotation studies. Phys. Med. Biol..

[24] Yang C., Lei U. (2007). Quasistatic force and torque on ellipsoidal particles under generalized dielectrophoresis. J. Appl. Phys..

[25] Arai F., Maruyama H., Sakami T., Ichikawa A., Kouketsu N., Dong L., Fukuda T. In pinpoint injection of micro tools using dielectrophoresis and hydrophobic surface for minimally invasive separation of microbe. Proceedings of the the Fifteenth IEEE International Conference on Micro Electro Mechanical Systems.

[26] Clow A.L., Gaynor P.T., Oback B.J. (2010). A novel micropit device integrates automated cell positioning by dielectrophoresis and nuclear transfer by electrofusion. Biomed. Microdevices.

[27] MacQueen L.A., Buschmann M.D., Wertheimer M.R. (2008). Gene delivery by electroporation after dielectrophoretic positioning of cells in a non-uniform electric field. Bioelectrochemistry.

[28] Tseng H.-Y., Huang Y.-H., Huang H.-Y., Yao D.-J. In oviduct-mimetic chip for sperm separation and oocyte manipulation to enhance the probability of fertilization for oligozoospermia patient. Proceedings of the 2013 8th IEEE International Conference on Nano/Micro Engineered and Molecular Systems (NEMS).

[29] Tuukkanen S., Kuzyk A., Toppari J., Hytönen V., Ihalainen T., Törmä P. (2005). Dielectrophoresis of nanoscale double-stranded DNA and humidity effects on its electrical conductivity. Appl. Phys. Lett..

[30] Srivastava S.K., Daggolu P.R., Burgess S.C., Minerick A.R. (2008). Dielectrophoretic characterization of erythrocytes: Positive abo blood types. Electrophoresis.

[31] Ramón-Azcón J., Yasukawa T., Mizutani F. (2010). Sensitive and spatially multiplexed detection system based on dielectrophoretic manipulation of DNA-encoded particles used as immunoreactions platform. Anal. Chem..

[32] Lee H.J., Lee S.H., Yasukawa T., Ramón-Azcón J., Mizutani F., Ino K., Shiku H., Matsue T. (2010). Rapid and simple immunosensing system for simultaneous detection of tumor markers based on negative-dielectrophoretic manipulation of microparticles. Talanta.

[33] Chuang C.-H., Ju J.-W., Huang Y.-W. (2013). Enhancing fluorescent response of immunosensing by a dielectrophoresis chip with transparent electrodes and microcavities array. Micro Nano Lett..

[34] Gascoyne P.R., Vykoukal J.V. (2004). Dielectrophoresis-based sample handling in general-purpose programmable diagnostic instruments. Proc. IEEE.

[35] Vykoukal J., Vykoukal D.M., Freyberg S., Alt E.U., Gascoyne P.R. (2008). Enrichment of putative stem cells from adipose tissue using dielectrophoretic field-flow fractionation. Lab. Chip.

[36] Voldman J. (2001). A Microfabricated Dielectrophoretic Trapping Array for Cell-Based Biological Assays. Ph.D. Thesis.

[37] Johari J., Hübner Y., Hull J.C., Dale J.W., Hughes M.P. (2003). Dielectrophoretic assay of bacterial resistance to antibiotics. Phys. Med. Biol..

[38] Chugh D., Kaler K.V.I.S. (2010). Integrated liquid and droplet dielectrophoresis for biochemical assays. Microfluidics Nanofluidics.

[39] Kato M., Sasamori E., Chiba T., Hanyu Y. (2011). Cell activation by CPG ODN leads to improved electrofusion in hybridoma production. J. Immunol. Methods.

[40] Tan C., Dannull J., Nair S.K., Ding E., Tyler D.S., Pruitt S.K., Lee W.T. (2013). Local secretion of IL-12 augments the therapeutic impact of dendritic cell–tumor cell fusion vaccination. J. Surg. Res..

[41] Cavallaro D., Capaccioli S., Carloni V. (2012). 147 targeting mechanism of cell fusion as a novel approach to abrogate multi-drug resistance of metastatic colon cancer. Eur. J. Cancer.

[42] Yang W.J., Li S.H., Weisel R.D., Liu S.M., Li R.K. (2012). Cell fusion contributes to the rescue of apoptotic cardiomyocytes by bone marrow cells. J. Cell. Mol. Med..

[43] Regtmeier J., Duong T.T., Eichhorn R., Anselmetti D., Ros A. (2007). Dielectrophoretic manipulation of DNA: Separation and polarizability. Anal. Chem..

[44] Huang Y., Joo S., Duhon M., Heller M., Wallace B., Xu X. (2002). Dielectrophoretic cell separation and gene expression profiling on microelectronic chip arrays. Anal. Chem..

[45] Sonnenberg A., Marciniak J.Y., Skowronski E.A., Manouchehri S., Rassenti L., Ghia E.M., Widhopf G.F., Kipps T.J., Heller M.J. (2014). Dielectrophoretic isolation and detection of cancer-related circulating cell-free DNA biomarkers from blood and plasma. Electrophoresis.

[46] Hu X., Bessette P.H., Qian J., Meinhart C.D., Daugherty P.S., Soh H.T. (2005). Marker-specific sorting of rare cells using dielectrophoresis. Proc. Natl. Acad. Sci. USA.

[47] Fu A.Y., Chou H.-P., Spence C., Arnold F.H., Quake S.R. (2002). An integrated microfabricated cell sorter. Anal. Chem..

[48] Moon H.-S., Kwon K., Kim S.-I., Han H., Sohn J., Lee S., Jung H.-I. (2011). Continuous separation of breast cancer cells from blood samples using multi-orifice flow fractionation (MOFF) and dielectrophoresis (DEP). Lab. Chip.

[49] Mohamad A., Jeynes J., Hughes M. (2014). Dielectrophoretic response of DNA shows different conduction mechanisms for poly (dg)-poly (dc) and poly (da)-poly (dt) in solution. NanoBiosci. IEEE Trans..

[50] Liang X., Graham K., Johannessen A., Costea D., Labeed F. (2014). Human oral cancer cells with increasing tumorigenic abilities exhibit higher effective membrane capacitance. Integr. Biol..

[51] Hölzel R., Calander N., Chiragwandi Z., Willander M., Bier F.F. (2005). Trapping single molecules by dielectrophoresis. Phys. Rev. Lett..

[52] Kumemura M., Collard D., Sakaki N., Yamahata C., Hosogi M., Hashiguchi G., Fujita H. (2011). Single-DNA-molecule trapping with silicon nanotweezers using pulsed dielectrophoresis. J. Micromech. Microeng..

[53] Chou C.-F., Tegenfeldt J.O., Bakajin O., Chan S.S., Cox E.C., Darnton N., Duke T., Austin R.H. (2002). Electrodeless dielectrophoresis of single-and double-stranded DNA. Biophys. J..

[54] Lapizco-Encinas B.H., Ozuna-Chacón S., Rito-Palomares M. (2008). Protein manipulation with insulator-based dielectrophoresis and direct current electric fields. J. Chromatogr. A.

[55] Thwar P.K., Linderman J.J., Burns M.A. (2007). Electrodeless direct current dielectrophoresis using reconfigurable field-shaping oil barriers. Electrophoresis.

[56] Shafiee H., Caldwell J.L., Sano M.B., Davalos R.V. (2009). Contactless dielectrophoresis: A new technique for cell manipulation. Biomed. Microdevices.

[57] Jen C.-P., Chen T.-W. (2009). Trapping of cells by insulator-based dielectrophoresis using open-top microstructures. Microsyst. Technol..

[58] Jen C.-P., Chen T.-W. (2009). Selective trapping of live and dead mammalian cells using insulator-based dielectrophoresis within open-top microstructures. Biomed. Microdevices.

[59] Jang L.-S., Huang P.-H., Lan K.-C. (2009). Single-cell trapping utilizing negative dielectrophoretic quadrupole and microwell electrodes. Biosens. Bioelectron..

[60] Lan K.-C., Jang L.-S. (2011). Integration of single-cell trapping and impedance measurement utilizing microwell electrodes. Biosens. Bioelectron..

[61] Wang C.-C., Lan K.-C., Chen M.-K., Wang M.-H., Jang L.-S. (2013). Adjustable trapping position for single cells using voltage phase-controlled method. Biosens. Bioelectron..

[62] Hunt T., Westervelt R. (2006). Dielectrophoresis tweezers for single cell manipulation. Biomed. Microdevices.

[63] Kodama T., Osaki T., Kawano R., Kamiya K., Miki N., Takeuchi S. (2013). Round-tip dielectrophoresis-based tweezers for single micro-object manipulation. Biosens. Bioelectron..

[64] Higginbotham S.N., Sweatman D.R. (2008). A combined travelling wave dielectrophoresis and impedance sensing device for sensing biological cell suspensions. J. Phys. D.

[65] Hirota K., Inagaki S., Hamada R., Ishihara K., Miyake Y. (2014). Evaluation of a rapid oral bacteria quantification system using dielectrophoresis and the impedance measurement. Biocontrol Sci..

[66] Moon H.-S., Im H.T., Choi A., Jung H.-I. (2010). Real-time detection of food-borne bacterial adenosine triphosphate (ATP) using dielectrophoretic force and a bioluminescence sensor. Microchim. Acta.

[67] Yang L. (2009). Dielectrophoresis assisted immuno-capture and detection of foodborne pathogenic bacteria in biochips. Talanta.

[68] Menachery A., Kremer C., Wong P.E., Carlsson A., Neale S.L., Barrett M.P., Cooper J.M. (2012). Counterflow dielectrophoresis for trypanosome enrichment and detection in blood. Sci. Rep..

[69] Hamada R., Takayama H., Shonishi Y., Mao L., Nakano M., Suehiro J. (2013). A rapid bacteria detection technique utilizing impedance measurement combined with positive and negative dielectrophoresis. Sens. Actuators B.

[70] Li S., Cui H., Yuan Q., Wu J., Wadhwa A., Eda S., Jiang H. (2014). AC electrokinetics-enhanced capacitive immunosensor for point-of-care serodiagnosis of infectious diseases. Biosens. Bioelectron..

[71] Baek S.H., Chang W.-J., Baek J.-Y., Yoon D.S., Bashir R., Lee S.W. (2009). Dielectrophoretic technique for measurement of chemical and biological interactions. Anal. Chem..

[72] Park I.S., Eom K., Son J., Chang W.-J., Park K., Kwon T., Yoon D.S., Bashir R., Lee S.W. (2012). Microfluidic multifunctional probe array dielectrophoretic force spectroscopy with wide loading rates. ACS Nano.

[73] Huang C., Liu H., Bander N.H., Kirby B.J. (2013). Enrichment of prostate cancer cells from blood cells with a hybrid dielectrophoresis and immunocapture microfluidic system. Biomed. Microdevices.

[74] Huang C.-T., Amstislavskaya T.G., Chen G.-H., Chang H.-H., Chen Y.-H., Jen C.-P. (2013). Selectively concentrating cervical carcinoma cells from red blood cells utilizing dielectrophoresis with circular ito electrodes in stepping electric fields. J. Med. Biol. Eng..

[75] Chen D., Du H., Tay C.Y. (2010). Rapid concentration of nanoparticles with DC dielectrophoresis in focused electric fields. Nanoscale Res. Lett..

[76] Chen D., Du H. (2010). A microfluidic device for rapid concentration of particles in continuous flow by DC dielectrophoresis. Microfluidics Nanofluidics.

[77] Cho Y.K., Kim S., Lee K., Park C., Lee J.G., Ko C. (2009). Bacteria concentration using a membrane type insulator-based dielectrophoresis in a plastic chip. Electrophoresis.

[78] Gallo-Villanueva R.C., Rodríguez-López C.E., Díaz-de-la-Garza R.I., Reyes-Betanzo C., Lapizco-Encinas B.H. (2009). DNA manipulation by means of insulator-based dielectrophoresis employing direct current electric fields. Electrophoresis.

[79] Lewpiriyawong N., Yang C., Lam Y.C. (2008). Dielectrophoretic manipulation of particles in a modified microfluidic h filter with multi-insulating blocks. Biomicrofluidics.

[80] Zhu J., Xuan X. (2009). Dielectrophoretic focusing of particles in a microchannel constriction using dc-biased AC flectric fields. Electrophoresis.

[81] Lewpiriyawong N., Yang C., Lam Y.C. (2012). Electrokinetically driven concentration of particles and cells by dielectrophoresis with dc-offset AC electric field. Microfluidics Nanofluidics.

[82] Hu N., Yang J., Yin Z.Q., Ai Y., Qian S., Svir I.B., Xia B., Yan J.W., Hou W.S., Zheng X.L. (2011). A high-throughput dielectrophoresis-based cell electrofusion microfluidic device. Electrophoresis.

[83] Hu N., Yang J., Qian S., Joo S.W., Zheng X. (2011). A cell electrofusion microfluidic device integrated with 3D thin-film microelectrode arrays. Biomicrofluidics.

[84] Tresset G., Takeuchi S. (2004). A microfluidic device for electrofusion of biological vesicles. Biomed. Microdevices.

[85] Gel M., Suzuki S., Kimura Y., Kurosawa O., Techaumnat B., Oana H., Washizu M. (2009). Microorifice-based high-yield cell fusion on microfluidic chip: Electrofusion of selected pairs and fusant viability. NanoBiosci. IEEE Trans..

[86] Kimura Y., Gel M., Techaumnat B., Oana H., Kotera H., Washizu M. (2011). Dielectrophoresis-assisted massively parallel cell pairing and fusion based on field constriction created by a micro-orifice array sheet. Electrophoresis.

[87] Şen M., Ino K., Ramón-Azcón J., Shiku H., Matsue T. (2013). Cell pairing using a dielectrophoresis-based device with interdigitated array electrodes. Lab. Chip.

[88] Elitas M., Martinez-Duarte R., Dhar N., McKinney J.D., Renaud P. (2014). Dielectrophoresis-based purification of antibiotic-treated bacterial subpopulations. Lab. Chip.

[89] Chen C.-C., Lin P.-H., Chung C.-K. (2014). Microfluidic chip for plasma separation from undiluted human whole blood samples using low voltage contactless dielectrophoresis and capillary force. Lab. Chip.

[90] Voldman J., Braff R.A., Toner M., Gray M.L., Schmidt M.A. (2001). Holding forces of single-particle dielectrophoretic traps. Biophys. J..

[91] Li H., Zheng Y., Akin D., Bashir R. (2005). Characterization and modeling of a microfluidic dielectrophoresis filter for biological species. Microelectromech. Syst. J..

[92] Morgan H., Hughes M.P., Green N.G. (1999). Separation of submicron bioparticles by dielectrophoresis. Biophys. J..

[93] Gascoyne P., Mahidol C., Ruchirawat M., Satayavivad J., Watcharasit P., Becker F.F. (2002). Microsample preparation by dielectrophoresis: Isolation of malaria. Lab. Chip.

[94] Li H., Bashir R. (2002). Dielectrophoretic separation and manipulation of live and heat-treated cells of listeria on microfabricated devices with interdigitated electrodes. Sens. Actuators B.

[95] Arnold W.M. (2001). Positioning and levitation media for the separation of biological cells. Ind. Appl. IEEE Trans..

[96] Choi W., Kim J.-S., Lee D.-H., Lee K.-K., Koo D.-B., Park J.-K. (2008). Dielectrophoretic oocyte selection chip for *in vitro* fertilization. Biomed. Microdevices.

[97] Yu L., Iliescu C., Xu G., Tay F.E. (2007). Sequential field-flow cell separation method in a dielectrophoretic chip with 3-d electrodes. Microelectromech. Syst. J..

[98] Cemazar J., Vrtacnik D., Amon S., Kotnik T. (2011). Dielectrophoretic field-flow microchamber for separation of biological cells based on their electrical properties. NanoBiosci. IEEE Trans..

[99] Gascoyne P.R., Noshari J., Anderson T.J., Becker F.F. (2009). Isolation of rare cells from cell mixtures by dielectrophoresis. Electrophoresis.

[100] Piacentini N., Mernier G., Tornay R., Renaud P. (2011). Separation of platelets from other blood cells in continuous-flow by dielectrophoresis field-flow-fractionation. Biomicrofluidics.

[101] Lee J., Kim Y., Beebe D.J., Kim B. (2012). Harnessing gravitational, hydrodynamic and negative dielectrophoretic forces for higher throughput cell sorting. BioChip J..

[102] Kralj J.G., Lis M.T., Schmidt M.A., Jensen K.F. (2006). Continuous dielectrophoretic size-based particle sorting. Anal. Chem..

[103] Han K.-H., Han S.-I., Frazier A.B. (2009). Lateral displacement as a function of particle size using a piecewise curved planar interdigitated electrode array. Lab. Chip.

[104] Kim U., Qian J., Kenrick S.A., Daugherty P.S., Soh H.T. (2008). Multitarget dielectrophoresis activated cell sorter. Anal. Chem..

[105] Han S.-I., Lee S.-M., Joo Y.-D., Han K.-H. (2011). Lateral dielectrophoretic microseparators to measure the size distribution of blood cells. Lab. Chip.

[106] Lapizco-Encinas B.H., Simmons B.A., Cummings E.B., Fintschenko Y. (2004). Insulator-based dielectrophoresis for the selective concentration and separation of live bacteria in water. Electrophoresis.

[107] Lapizco-Encinas B.H., Simmons B.A., Cummings E.B., Fintschenko Y. (2004). Dielectrophoretic concentration and separation of live and dead bacteria in an array of insulators. Anal. Chem..

[108] Jen C.-P., Huang C.-T., Shih H.-Y. (2010). Hydrodynamic separation of cells utilizing insulator-based dielectrophoresis. Microsyst. Technol..

[109] Kang K.H., Kang Y., Xuan X., Li D. (2006). Continuous separation of microparticles by size with direct current-dielectrophoresis. Electrophoresis.

[110] Kang Y., Li D., Kalams S.A., Eid J.E. (2008). Dc-dielectrophoretic separation of biological cells by size. Biomed. Microdevices.

[111] Zhang L., Tatar F., Turmezei P., Bastemeijer J., Mollinger J., Piciu O., Bossche A. (2006). Continuous electrodeless dielectrophoretic separation in a circular channel. J. Phys..

[112] Viefhues M., Eichhorn R., Fredrich E., Regtmeier J., Anselmetti D. (2012). Continuous and reversible mixing or demixing of nanoparticles by dielectrophoresis. Lab. Chip.

[113] Viefhues M., Regtmeier J., Anselmetti D. (2013). Fast and continuous-flow separation of DNA-complexes and topological DNA variants in microfluidic chip format. Analyst.

[114] Viefhues M., Wegener S., Rischmüller A., Schleef M., Anselmetti D. (2013). Dielectrophoresis based continuous-flow nano sorter: Fast quality control of gene vaccines. Lab. Chip.

[115] Lei U., Huang C., Chen J., Yang C., Lo Y., Wo A., Chen C., Fung T. (2009). A travelling wave dielectrophoretic pump for blood delivery. Lab. Chip.

[116] Van den Driesche S., Rao V., Puchberger-Enengl D., Witarski W., Vellekoop M.J. (2012). Continuous cell from cell separation by traveling wave dielectrophoresis. Sens. Actuators B.

[117] Cheng I.-F., Froude V.E., Zhu Y., Chang H.-C., Chang H.-C. (2009). A continuous high-throughput bioparticle sorter based on 3d traveling-wave dielectrophoresis. Lab. Chip.

[118] Choi E., Kim B., Park J. (2009). High-throughput microparticle separation using gradient traveling wave dielectrophoresis. J. Micromech. Microeng..

[119] Zhao Y., Yi U.-C., Cho S.K. (2007). Microparticle concentration and separation by traveling-wave dielectrophoresis (twDEP) for digital microfluidics. Microelectromech. Syst. J..

[120] Zhao Y., Yi U.-C., Cho S.K. In Highly efficient in-droplet particle concentration and separation by twDEP and EWOD for digital microfluidics. Proceedings of IEEE 20th International Conference on MEMS.

[121] Wang L., Lu J., Marchenko S.A., Monuki E.S., Flanagan L.A., Lee A.P. (2009). Dual frequency dielectrophoresis with interdigitated sidewall electrodes for microfluidic flow-through separation of beads and cells. Electrophoresis.

[122] Vahey M., Voldman J. (2008). An equilibrium method for continuous-flow cell sorting using dielectrophoresis. Anal. Chem..

[123] Markx G.H., Carney L., Littlefair M., Sebastian A., Buckle A.-M. (2009). Recreating the hematon: Microfabrication of artificial haematopoietic stem cell microniches *in vitro* using dielectrophoresis. Biomed. Microdevices.

[124] Gray D.S., Tan J.L., Voldman J., Chen C.S. (2004). Dielectrophoretic registration of living cells to a microelectrode array. Biosens. Bioelectron..

[125] Hakoda M., Hirota Y. (2013). Correlation between dielectric property by dielectrophoretic levitation and growth activity of cells exposed to electric field. Bioprocess. Biosyst. Eng..

[126] Park K., Suk H.-J., Akin D., Bashir R. (2009). Dielectrophoresis-based cell manipulation using electrodes on a reusable printed circuit board. Lab. Chip.

[127] Donato S.S., Chu V., Prazeres D.M., Conde J.P. (2013). Metabolic viability of *Escherichia coli* trapped by dielectrophoresis in microfluidics. Electrophoresis.

[128] Jaeger M., Mueller T., Schnelle T. (2007). Thermometry in dielectrophoresis chips for contact-free cell handling. J. Phys. D.

[129] Li M., Li S., Cao W., Li W., Wen W., Alici G. (2013). Improved concentration and separation of particles in a 3D dielectrophoretic chip integrating focusing, aligning and trapping. Microfluidics Nanofluidics.

[130] Tay F.E., Yu L., Pang A.J., Iliescu C. (2007). Electrical and thermal characterization of a dielectrophoretic chip with 3D electrodes for cells manipulation. Electrochim. Acta.

